# Supplementary figures and images for: Investigation of the genetic diversity of gut mycobiota of the wild and laboratory mice
Source: Microbiol Spectr. 2025 Mar 31;13(5):e02840-24. doi: 10.1128/spectrum.02840-24 (PMC12054021; doi:10.1128/spectrum.02840-24)

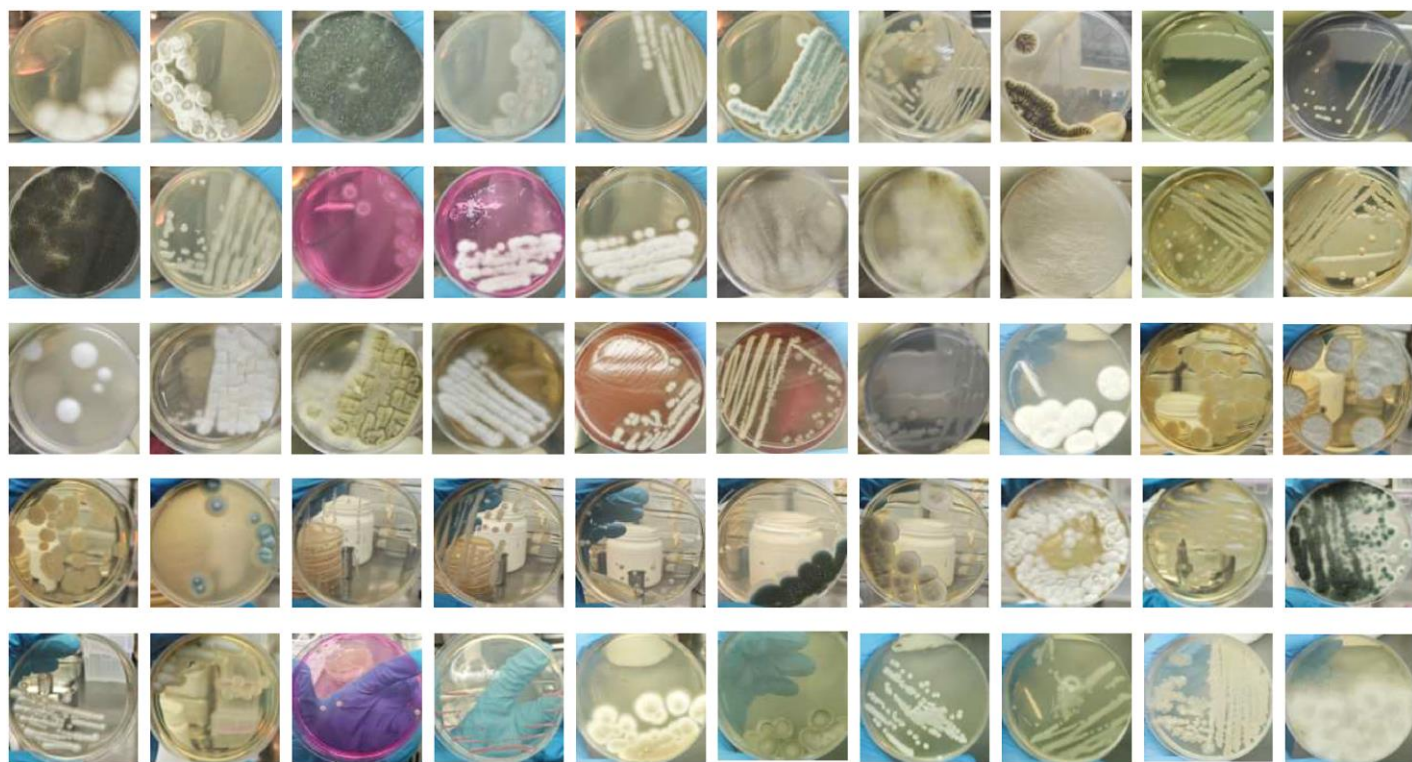

**Supplementary Figure S1:** Strain morphology of the cultured fungi

Supplement: Figure S1 — Strain morphology of the cultured fungi. [file spectrum.02840-24-s0001.pdf]
