# Supplementary material for: Investigation of the genetic diversity of gut mycobiota of the wild and laboratory mice
Source: Microbiol Spectr. 2025 Mar 31;13(5):e02840-24. doi: 10.1128/spectrum.02840-24 (PMC12054021; doi:10.1128/spectrum.02840-24)
Supplement: Figure S2 — Species composition at order level and comparative genome analysis. [file spectrum.02840-24-s0002.pdf]

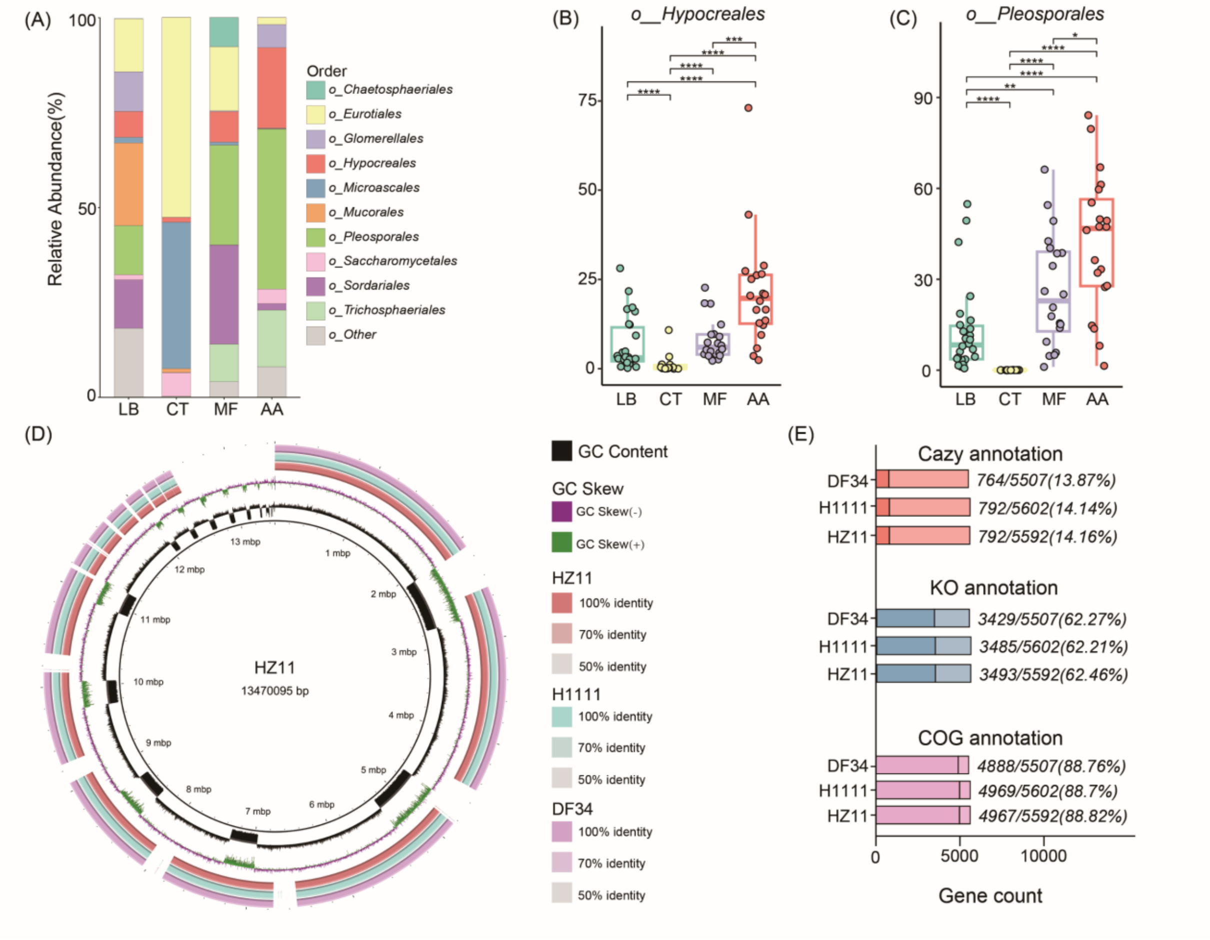

Supplementary Figure S2: Species composition at order level and comparative genome analysis: (**A**) Bar plots display community composition of the fungal microbiota at the order level among the four mouse species. (**B-C**) Boxplots show the relative abundance of *Hypocreales* and *Pleosporales* among the different mouse groups. (**D**) Comparison of conspecific genomes (*Meyerozyma guilliermondii*) isolated from *Microtus fortis* (DF34), *Lasiopodomys brandtii* (H1111) and C57BL/6J (HZ11) mice. The color intensity in each ring represents the BLAST match identity. (**E**) Stacked histograms of the gene count annotated CAZymes, COGs and KOs for three genomes, respectively. Values shown are the numbers of the annotated and total genes. Dark colors represent annotation, and light colors denote no annotation.
